# Silica, Silicosis, and Autoimmunity

**DOI:** 10.3389/fimmu.2016.00097

**Published:** 2016-03-11

**Authors:** Kenneth Michael Pollard

**Affiliations:** ^1^Department of Molecular and Experimental Medicine, The Scripps Research Institute, La Jolla, CA, USA

**Keywords:** silica, silicosis, autoimmunity, human, animal model

## Abstract

Inhalation of dust containing crystalline silica is associated with a number of acute and chronic diseases including systemic autoimmune diseases. Evidence for the link with autoimmune disease comes from epidemiological studies linking occupational exposure to crystalline silica dust with the systemic autoimmune diseases systemic lupus erythematosus, systemic sclerosis, and rheumatoid arthritis. Although little is known regarding the mechanism by which silica exposure leads to systemic autoimmune disease, there is a voluminous literature on silica exposure and silicosis that may help identify immune processes that precede development of autoimmunity. The pathophysiology of silicosis consists of deposition of silica particles in the alveoli of the lung. Ingestion of these particles by macrophages initiates an inflammatory response, which stimulates fibroblasts to proliferate and produce collagen. Silica particles are encased by collagen leading to fibrosis and the nodular lesions characteristic of the disease. The steps in the development of silicosis, including acute and chronic inflammation and fibrosis, have different molecular and cellular requirements, suggesting that silica-induced inflammation and fibrosis may be mechanistically separate. Significantly, it is unclear whether silica-induced inflammation and fibrosis contribute similarly to the development of autoimmunity. Nonetheless, the findings from human and animal model studies are consistent with an autoimmune pathogenesis that begins with activation of the innate immune system leading to proinflammatory cytokine production, pulmonary inflammation leading to activation of adaptive immunity, breaking of tolerance, and autoantibodies and tissue damage. The variable frequency of these immunological features following silica exposure suggests substantial genetic involvement and gene/environment interaction in silica-induced autoimmunity. However, numerous questions remain unanswered.

## Introduction

Environmental factors play a significant role in the development of human autoimmunity (1). These factors include the food we eat, the fluids we drink, the air we breathe, chemicals (natural and synthetic), infections, by-products of manufacturing processes, and radiation (2–4). A recent review of the epidemiologic evidence of environmental factors in human autoimmune diseases concluded that exposure to crystalline silica contributes to the development of a number of autoimmune diseases, including systemic lupus erythematosus (SLE), rheumatoid arthritis (RA), systemic sclerosis (SSc), and antineutrophil cytoplasmic antibody (ANCA)-related vasculitis (5). Despite this strong linkage of silica exposure with autoimmune diseases, there is little evidence of the possible mechanisms underlying this relationship (6, 7). This is due in large part to a lack of accepted criteria for diagnosis or classification of environmentally associated autoimmunity (8) as well as a paucity of animal models that mimic features of silica exposure in humans (6). In contrast, there is a voluminous literature on silica exposure and the development of silicosis in humans and animal models (9–11). In this article, I provide a brief overview of the immunological consequences of silica exposure and discuss how an understanding of identified mechanisms and biological markers may contribute to an understanding of silica-induced autoimmunity.

## Silica and Inflammation

Silica (SiO_2_) is an oxide of silicon and is most commonly found in nature as quartz. Silica exists in many crystalline forms (called polymorphs) with α-quartz being the most common form (11). Exposure to respirable crystalline silica (<10 μm in size) occurs most often in occupational settings, where materials containing crystalline silica are reduced to dust or when fine particles are disturbed. These occupations are often called the dusty trades and include abrasive blasting with sand, jack hammering, drilling, mining/tunneling operations, and cutting and sawing (10, 12). Inhaling crystalline silica dust can lead to silicosis, bronchitis, or cancer (10, 11). Silicosis is characterized by chronic inflammation and scarring in the upper lobes of the lungs and can be classified based on the quantity inhaled, time course, and length of exposure (10, 11, 13). Chronic simple silicosis is the most common form, occurring after 15–20 years of moderate to low exposures to respirable crystalline silica. The accelerated form occurs after 5–10 years of high exposures to respirable crystalline silica, and acute silicosis, or silicoproteinosis, occurs after a few months or as long as 5 years following exposures to extremely high concentrations of respirable crystalline silica. The acute form is the most severe form of silicosis. The pathophysiology of silicosis involves deposition of particles into alveoli where they cannot be cleared. Ingestion of these particles by alveolar macrophages initiates an inflammatory response, which stimulates fibroblasts to proliferate and produce collagen. The silica particles are enveloped by collagen leading to fibrosis and nodular lesions characteristic of the disease.

## Cell and Molecular Requirements for Silica-Induced Inflammation/Fibrosis

**Figure 1:**
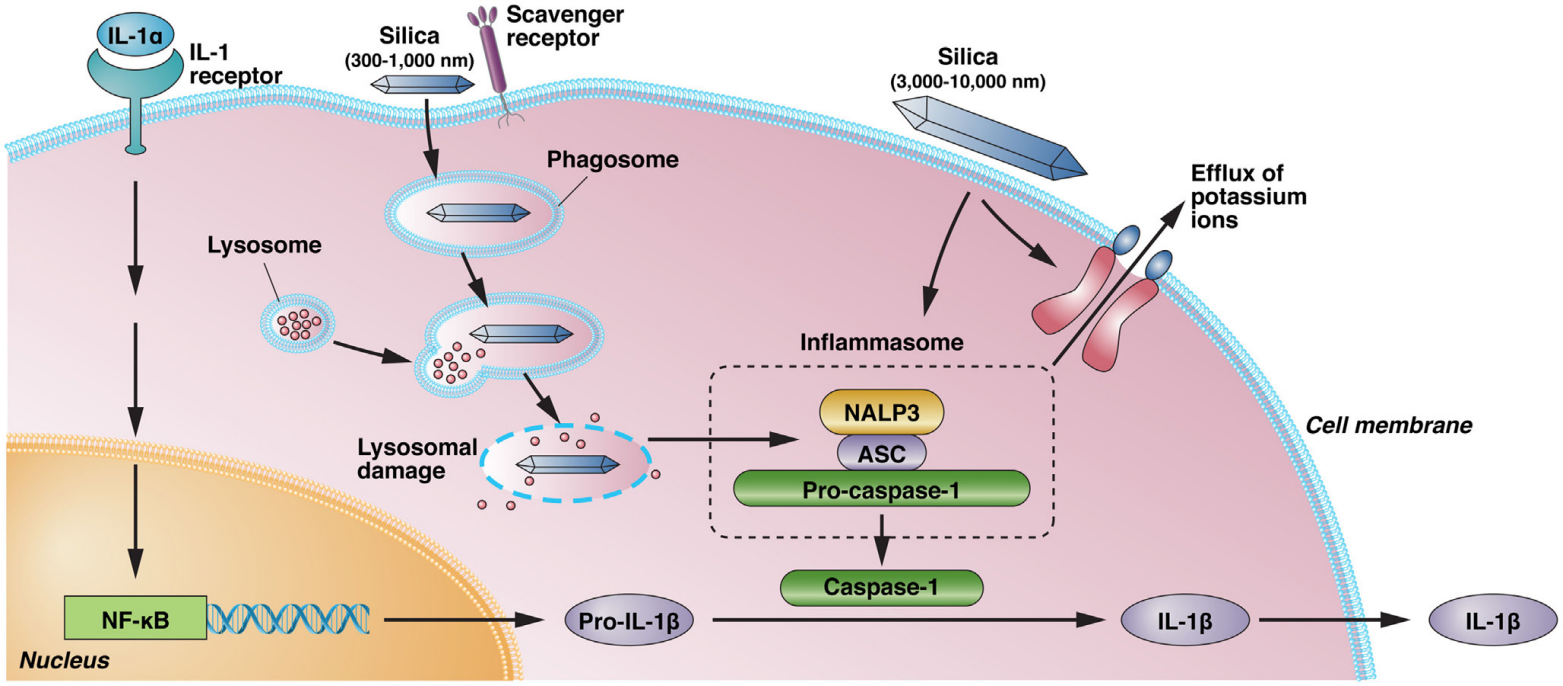
**Silica-induced activation of inflammasome and IL-1 production**. IL-1α, released from alveolar macrophages following crystalline exposure, results in NF-κB activation and transcription and translation of pro-IL-1β. Phagocytosis of crystalline silica leads to phagosomal damage and release of phagosome contents into the cytoplasm. This results in the activation of NALP3 and its association with the intracellular adapter protein ASC, which combines with and activates pro-caspase-1. The resulting inflammasome cleaves pro-IL-1β to the proinflammatory IL-1β. However, binding of immobilized silica crystals to the cell membrane of macrophages is also sufficient to induce IL-1β without evidence of lysosomal damage. Activation of the NALP3 inflammasome by silica also results in efflux of intracellular potassium ions, suggesting a possible interaction of silica with a membrane-associated protein, but it is unclear if K^+^ efflux following binding of immobilized silica crystals to the cell membrane results in inflammasome activation. Scavenger receptors have a role in the recognition and uptake of silica. NALP3, NACHT, LRR, and PYD domains-containing protein 3; ASC, apoptosis-associated speck-like protein containing a caspase recruitment domain; NF-κB, nuclear factor-κB; IL, interleukin.

A number of studies in experimental animals have revealed differences in silica-induced inflammatory responses and silicosis (14–16), arguing that gene–environment interactions are important in the severity of disease. Gene deletion studies have identified a number of the cellular and molecular requirements for silica-induced inflammation and fibrosis. The inflammatory response following exposure to crystalline silica is mediated by NALP3 inflammasome-driven IL-1β (17). Inflammasome activation is argued to occur following uptake of silica by scavenger receptors, lysosomal rupture, and release of cathepsin B accompanied by production of reactive oxygen species (ROS) and potassium efflux (10, 17–19) (Figure 1). The binding of silica to scavenger receptors also results in apoptosis of macrophages and the release of mediators (e.g., proinflammatory cytokines) contributing to lung inflammation and fibrosis (20). However, scavenger receptors also play a significant role in clearing silica, and their absence enhances inflammation but not fibrosis (21, 22).

**Figure 2:**
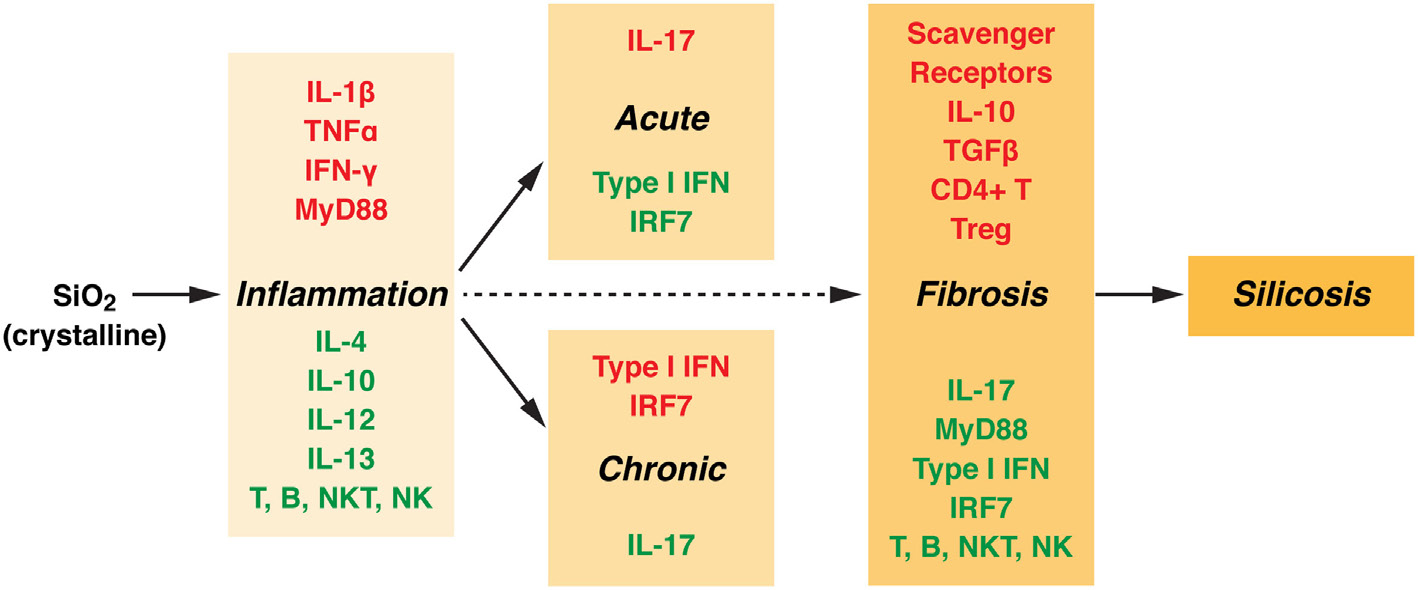
**Molecular and cellular components involved in silica-induced inflammation and fibrosis**. Silicosis is marked by inflammation and fibrosis with the formation of nodular lesions in the upper lobes of the lungs. The collagen containing silicotic nodules are a specific response to crystalline silica. However, the cellular and molecular components responsible for the inflammatory and fibrotic responses are not the same. Components required for inflammation (acute or chronic) and fibrosis are highlighted in red, while those not essential have been highlighted in green (see text for details). TNF, tumor necrosis factor; IFN, interferon; MyD88, myeloid differentiation primary response gene 88; IL, interleukin; NK, natural killer; NKT, natural killer T cell; IRF, interferon regulatory factor; TGF, transforming growth factor.

Consistent with the differential requirements for scavenger receptors, the steps in the development of silicosis, including acute and chronic inflammation and fibrosis, have different molecular and cellular requirements (Figure 2). Inflammation and fibrosis occur independently of T, B, NKT, and NK cells (23), although treatment with anti-CD4 antibodies reduces the severity of fibrosis (24). This may be explained by the presumptive role of T regulatory cells in fibrosis (25). Deficiency of IL-1α reduced IL-1β production and neutrophil accumulation following silica exposure (26), suggesting that release of endogenous IL-1α from alveolar macrophages promotes subsequent lung inflammation. Pulmonary inflammation is also dependent on IFN-γ (27), but not IL-4 or IL-13 (28) or IL-12 (29). In keeping with its anti-inflammatory potential, IL-10 helps limit the silica-induced inflammatory response but amplifies the fibrotic response (30). The role of IL-10 in fibrosis appears to be due to exacerbation of the Th2 response and the production of profibrotic IL-4 and IL-13 (31). Acute inflammation, but not chronic inflammation or fibrosis, requires IL-17 (32), conversely, chronic inflammation, but not acute inflammation or fibrosis, requires type I IFN and IRF7 (33). Additional studies have suggested that presence of innate immune response components (particularly IL-1 receptor, IL-1, ASC, NALP3, IL-18 receptor, IL-33 receptor, TRIF, and TLR2, 3, and 4) are not required for accumulation of collagen in the lung (fibrosis), while inflammation, neutrophil accumulation, IL-1β release, and granuloma formation did require MyD88 (25). In contrast, others have suggested that absence of NALP3 and ASC reduces collagen deposition (17). While these studies may question the role of innate immunity in fibrosis, it is becoming clear that silica-induced inflammation and fibrosis can be uncoupled as evidenced by the observation that steroid treatment reduced lung inflammation and proinflammatory cytokine expression (TNF-α, IL-1β) but had no significant effect on lung fibrosis or expression of fibrogenic cytokines (TGF-β and IL-10) (34).

## Properties of Silica That Influence Inflammation

Both amorphous (non-crystalline) silica particles and crystalline silica are phagocytosed by, and toxic to, macrophages leading to endolysosomal rupture and caspase-3 activation (35). Nevertheless, the size of silica particles can dramatically affect the inflammatory response. Amorphous silica particles of 30–1,000 nm in diameter induce greater inflammatory responses, as judged by lysosomal destabilization, proinflammatory cytokine expression, and pulmonary inflammation, than 3,000–10,000 nm particles (36). However, it is unclear if silica-induced lysosome destabilization is essential to NLRP3 inflammasome activation, IL-1β production, and inflammation. The binding of immobilized silica crystals to the cell membrane of macrophages was sufficient to induce IL-1β without evidence of lysosomal damage or a requirement for cathepsin B (18) (Figure 1). Blocking K^+^ efflux from the cell was sufficient to reduce IL-1β release although whether potassium efflux is directly responsible for NLRP3 activation remains unclear (18). An alternative explanation for silica-induced IL-1β expression argues that silica exposure results in release of IL-1α into the alveolar space, which then drives production of IL-1β and lung inflammation (26). This explanation is consistent with the concept that IL-1α functions as an alarm molecule and plays a critical role early in inflammation (37).

## Silica-Associated Autoimmunity

A number of epidemiological studies support the association between occupational exposure to respirable crystalline silica dust and development of systemic autoimmune diseases (5, 12). Exposure to asbestos, another silicate that occurs in mining and construction, may be concurrent with crystalline silica exposure. While it can be difficult to assess the role of each separately, epidemiological data are too limited to argue for a strong association between asbestos exposure and autoimmunity (5). However, there is growing evidence that asbestos exposure may be associated with autoimmunity (e.g., hypergammaglobulinemia and autoantibodies) in the absence of confirmed autoimmune disease (38, 39). This is an important observation as several studies of crystalline silica exposure also point to the appearance of features of autoimmunity, especially autoantibodies, in exposed individuals in the absence of autoimmune disease (40, 41). This suggests that study of larger cohorts of asbestos exposed individuals may lead to stronger associations with autoimmune diseases.

For respirable crystalline silica dust, the prevalence of disease is increased when compared to the general population and shows evidence of strong occupational bias mostly associated with males (41). In high-level exposure, SLE is 10 times higher than the expected sex-specific prevalence in the general population (12, 41), but the strength of this association falls in both men and women as exposure is reduced (42). Moreover, there is evidence that disease features may differ between those with silica-induced systemic autoimmune disease and those with idiopathic disease (41, 42). Uranium miners with SLE had considerably less arthritis and also less photosensitivity compared to those with idiopathic SLE (41); there was also reduced prevalence of discoid lesions although this did not reach statistical significance. However, the silica group was all males with late onset disease, while the idiopathic disease group was 90% females and matched only for geographical location and ethnicity. Thus, it is unclear if the differences reflect silica exposure or sex and/or age differences. In the second study, demographic characteristics were more carefully controlled; however, the silica-exposed SLE patients were found to have reduced prevalence of anemia and leukopenia (42). Differences in autoantibodies have also been reported. Patients with silica-associated SSc had greater prevalence of anti-DNA topoisomerase 1 autoantibodies, and both silica-associated SSc and SLE had fewer patients with high titer antinuclear antibodies (ANA) (>1:1,280) compared to those with idiopathic disease (40).

Individual study populations have been found to have increased occurrence of different diseases suggesting a common underlying pathophysiology (43). This is supported by the observation that clinical features and autoantibodies specific to connective tissue diseases including anti-DNA, anti-SS-A/Ro, anti-SS-B/La, anti-centromere, and anti-topoisomerase 1 occur at higher frequency in exposed individuals without autoimmune disease compared to the general population (12, 40, 42). Although silicosis may be associated with immune abnormalities including autoantibodies, the association of silica exposure with expression of autoimmune disease can occur in the absence of silicosis (12, 41). Furthermore, while there is an association between intensity of exposure and autoantibodies including an association of high-level exposure with SLE, there is no relationship between autoantibodies and silicosis (12, 41, 44). This suggests that the development of fibrosis and nodular lesions may not be required for development of autoimmunity. Whether this reflects the recent suggestion that fibrosis is linked to T regulatory cells (25) is uncertain.

The variable frequency of disease features in silica-induced autoimmunity suggests significant genetic involvement and gene/environment interactions. Silicosis can occur in 47–77% of individuals with adequate follow-up after silica exposure (45). In patients with silicosis, hypergammaglobulinemia can occur in over 65% of patients (46). In silicosis, ANA prevalence can be 34% or higher (47). End-stage renal disease due to silica exposure occurs in about 5% of exposed individuals (45), and development of diagnostically definable systemic autoimmune disease is even less frequent (12). These findings are consistent with a disease progression that begins with silica-induced activation of the innate immune system leading to inflammation of the lung, activation of adaptive immunity, breaking of tolerance, and autoantibodies and tissue damage.

## Animal Modeling of Silica Exposure to Mimic Human Autoimmunity

Only a small number of animal studies have modeled silica-induced autoimmunity. Lupus-prone NZM2410 mice exposed to crystalline silica exhibit pulmonary inflammation and fibrotic lesions, autoantibodies, kidney deposits of IgG and C3, proteinuria, and reduced survival compared to controls (48). A follow-up study reported increased TNF-α in bronchoalveolar lavage fluid (BALF), B1a B, and CD4^+^ T cells in lymph node as well as alteration in the ratio of CD4^+^ to CD4^+^CD25^+^ T cells (49). A more recent study using lupus-prone NZBWF1 mice confirmed the exacerbation of SLE-like disease as well as identifying the formation of inducible bronchus-associated lymphoid tissue (iBALT) (50). Exposure to asbestos induces a similar spectrum of autoantibodies, kidney immune deposits, and changes in CD4^+^CD25^+^ T cells in non-autoimmune prone C57BL/6 mice (51). Eronite, an asbestos-like fibrous mineral, induced ANA, IL-17, TNF-α, and renal deposits of IgG in C57BL/6 mice (52). Asbestos exposure in Lewis rats failed to exacerbate arthritis induced by collagen or peptidoglycan–polysaccharide but did induce ANA, anti-histidyl tRNA synthetase antibodies, and proteinuria but showed no evidence of kidney immune deposits (53, 54). Non-autoimmune Brown Norway rats given sodium silicate (NaSiO_4_) by subcutaneous injection developed ANA including anti-DNA, -Sm, -SS-A, and -SS-B (55). The ANA titers increased with time with the majority being positive for anti-RNP (56). These studies demonstrate that crystalline silica, and asbestos, can elicit autoimmunity in mice and rats and that non-crystalline silica can induce humoral autoimmunity in non-autoimmune prone rats, but they provide little evidence for possible mechanisms.

When mechanism has been examined, the results point to a significant role for cell death as a source of immune stimulation. ANA from crystalline silica-exposed NZM2410 mice preferentially bind to alveolar macrophage-like cells undergoing silica-induced apoptosis but not if apoptosis was inhibited by a caspase inhibitor (57), suggesting that silica-induced autoantibodies are directed against material from dying cells. This is supported by the observation that apoptosis induced by asbestos exposure results in surface blebs enriched in the autoantigen SSA/Ro52, which are bound by autoantibodies from asbestos-exposed mice (58). Rottlerin, which affects kinase and non-kinase proteins as well as activating K^+^ channels (59), reduced silica-induced proteinuria, autoantibodies, and IgG and C3 kidney deposits in lupus-prone NZM2410 mice (60). These studies led to the hypothesis that silica-induced activation of alveolar macrophages leads to apoptosis and inflammation, ingestion of cellular debris, migration of activated antigen-presenting cells (APCs) to lymph nodes, and activation of T and B cells (61). However, it is unclear how this leads to breaking of self-tolerance.

## Conclusion

In aggregate, the immunological consequences of silica exposure that lead to autoimmunity are consistent with a disease progression that begins with activation of the innate immune system resulting in proinflammatory cytokine production, inflammation of the lung leading to activation of adaptive immunity, breaking of tolerance, and autoantibodies and renal damage. However, numerous questions remain unanswered.

It is unknown if the early events leading to IL-1β expression (Figure 1) are required for silica-induced autoimmunity. Are there size, shape, surface area, or charge rules for silica-induced lysosomal destabilization, K^+^ efflux, and inflammasome activation? Does K^+^ efflux play a role in silica-induced inflammation/autoimmunity? The contribution of the inflammasome and IL-1 to systemic autoimmunity remains unclear (62) because while caspase 1 is required for pristane-induced autoimmunity (63), neither caspase 1 nor NALP3 is required for mercury-induced autoimmunity (64). Additional research is also needed to determine if nanoparticles and other non-crystalline forms of silica lead to autoimmunity.

Many of the genetic requirements for silica-induced inflammation (Figure 2) are also required for systemic autoimmunity. In particular, IFN-α/β and/or IFN-γ are required for idiopathic (65) and induced systemic autoimmunity (66, 67). Additionally, the role of IL-17 in autoimmunity continues to grow (68). Conversely, genetic elements required for silica-induced fibrosis may play little role in silica-induced autoimmunity. Deficiency of scavenger receptors exacerbates autoantibody responses (69). Moreover, the protective role of T regulatory cells and their cytokines IL-10 and TGF-β in systemic autoimmunity (70, 71) argues that the fibrotic process elicited by silica exposure may negatively regulate the development of autoimmunity. It remains to be determined which of the molecular and cellular components that drive silica-induced inflammation and fibrosis explain the variable frequency of immunological features found in silica-induced autoimmunity.

Finally, a significant concern for future research is the paucity of animal models of silica-induced autoimmunity (6). Susceptibility to silicosis varies among inbred mouse strains (15) and no single inbred mouse strain mimics the genetic or disease heterogeneity found in humans. Considerable effort will be needed to identify an appropriate experimental model so that studies can be “shaped by what is observed in humans, not by what is possible in mice” (72).

## Author Contributions

The author confirms being the sole contributor of this work and approved it for publication.

## Conflict of Interest Statement

The author declares that this manuscript and associated research was done in the absence of any commercial or financial relationships that could be construed as a potential conflict of interest.
